# Pride or backlash? Public attitudes towards the Spanish women’s national football team

**DOI:** 10.3389/fspor.2025.1727889

**Published:** 2025-12-02

**Authors:** David Moscoso-Sánchez, Sara Pasadas-del-Amo, Rodrigo Ramis-Moyano, Manuel Trujillo-Carmona

**Affiliations:** 1 Area de Sociología, Departamento de Ciencias Sociales, Filosofía, Geografía y Traducción e Interpretación, Universidad de Córdoba, Córdoba, Spain; 2 Institute for Advanced Social Studies, Spanish National Research Council, Córdoba, Spain

**Keywords:** antifeminism, athlete activism, FIFA women's world cup, gender equality, political polarisation, public attitudes, women's football

## Abstract

**Introduction:**

In August 2023, Spain's Women's National Football Team won the FIFA Women's World Cup for the first time. Beyond sporting success, this victory became a catalyst for intense public debate on gender equality and athlete activism. Shortly after the tournament, two events brought these issues to the forefront: the non-consensual kiss by the president of the Real Federación Española de Fútbol (RFEF), which triggered nationwide protests under the hashtag *#SeAcabó* (“It's over”), and a strike by players demanding better working conditions and higher wages. While these events deepened discussions about sexism in sport, they also revealed strong ideological divisions in Spanish society. This article examines how Spaniards perceived these events and the players’ labour activism.

**Methods:**

Drawing on nationally representative data from the October 2023 Barometer of the Centro de Investigaciones Sociológicas (CIS), we analyse support for the players’ demands and satisfaction with the team's victory. Using binary logistic regression models, we assess how sociodemographic and political factors shape these attitudes.

**Results:**

Results show broad public support for footballers’ demands but with marked ideological and gender divides. Women and left-wing citizens express stronger solidarity with the players, while right-wing and VOX voters are less supportive and less satisfied with the team's success.

**Discussion:**

These findings highlight how sport, gender, and politics intersect in Spain's contemporary public sphere and demonstrate that women's sport can serve both as a site of empowerment and a field of cultural and political contestation.

## 1 Introduction

The Spanish Women's National Football Team was undoubtedly the main protagonist of Spanish sport in 2023. Their victory in the FIFA Women's World Cup in August 2023 was a sporting event of national and international significance, as it marked the first world championship in the history of Spanish women's football. As such, this achievement received a great deal of media attention (1), placing the working conditions of female footballers and their efforts to achieve this historic milestone at the centre of public debate (2). In fact, participation in the competition was preceded by years of economic and labour demands by professional players (3), as well as by the resignation of fifteen players from the national team just one year before the World Cup, demanding changes (which did not occur) in the professional project (4, 5). However, the public debate was marked by two events that occurred just after the international championship and confronted the players with the country's main football institutions.

The first of these, the most widely reported incident, occurred during the awards ceremony after the World Cup final. The President of the RFEF^1^ (Luis Rubiales) kissed Jenni Hermoso (a player of the national team) on the lips without her consent, triggering a strong social and political outcry (4). Over the following days, there was a succession of public messages of support from her teammates. The hashtag *#SeAcabó* (“It's over”) quickly went viral on Twitter, resonating as a demand from Spanish society for accountability and changes on the part of institutions (6). Political parties also expressed their views on this sexist act, with VOX (radical right) being the only political party that did not support the player. Furthermore, Luis Rubiales himself, in his defending arguments, used anti-feminist arguments typical of the radical right (5).

The second event occurred just 20 days after the national team was crowned world champions. Coinciding with the start of the national women's league (September 2023), the players went on strike to demand better working conditions and an increase in their minimum wage, which at that time was a meagre €16,000 per year (about 11 times less than that of their male counterparts, €186,000). After several unsuccessful negotiations and under pressure from a prolonged strike, the employers' association representing the clubs accepted the demands (7). This context of ongoing conflict between the players and the main football institutions turned the sporting victory into a symbol of the struggle for gender equality, but also into an object of cultural and political dispute.

Although the activism of female players has been very present in the public debate surrounding women's football, little is known about how Spanish society perceives their demands and how this has influenced attitudes towards the Spanish Women's National Football Team. In fact, beyond the North American context, we know very little about social attitudes and preferences towards the activism of European female athletes (8). To address this gap, in this article we use data from Barometer No. 3423 (October 2023) from the Centro de Investigaciones Sociológicas (CIS) (9), the only data available on this subject. The barometer, conducted less than two months after the 2023 FIFA Women's World Cup, included several questions to survey citizens’ opinions on the national team's performance in this competition, as well as on the working conditions of its athletes and their demands. The aim was to ascertain the level of support that exists in Spain for women's football and its demands, as well as to examine the variables that influence Spanish citizens' views on this issue.

The results of the analyses show a high level of support for the female players. However, gender, ideological orientation, and vote recall predict lower support for the players' labour demands and lower satisfaction with the victory achieved in the Women's World Cup. Lower satisfaction with the sporting achievement is mainly observed among those who are less supportive of the players' demands. The analysis sheds light on the cultural dynamics shaping the attitudes towards female athletes' activism, raising new questions at the intersection between the sociology of sport and political sociology. It also highlights that women's sport can be an arena for cultural and political battles (10, 11). This is particularly relevant in Spain due to the growing ideological polarisation surrounding gender equality. Despite undeniable progress in this area in recent years, a parallel backlash has emerged, leading to anti-feminist discourse gradually gaining social traction, both among certain sectors of public opinion and across the political spectrum.

### 1.1 Women's sport and athlete activism: women's football in Spain

Sport has historically been a deeply masculinised space: not only because more men than women participate in it (both at amateur and, above all, professional levels), but also because it has functioned as a mechanism for reproducing the patriarchal order, associated with values such as strength, aggressiveness and hegemonic male competitiveness (11, 12)^2^. Women have long been deliberately marginalised from sporting spaces, with less institutional support and recognition than their male counterparts (13–15).

Football, one of the most popular sports in the world, is also one of the sports that most clearly shows these inequalities between men and women. Although women have been playing football since the 19th century and participated in large numbers in this sport in the early 20th century in some European countries (such as the United Kingdom, France and Norway), it was not until the last quarter of the last century that the process of (slow) consolidation of what is now the women's football scene began (16, 17)^3^. However, this process has not been and is not without obstacles. These range from the still limited presence of women in the main football institutions [e.g., (18)] to the precarious salaries and working conditions of female players [e.g., (19)], to unequal media coverage compared to men's football [e.g., (20)] and, ultimately, the pervasive reluctance of football institutions to support women's football [e.g., (21)]. This is not to mention other layers of discrimination such as traditional notions of femininity and prejudices about women's abilities, or the intersection of gender with other aspects such as sexuality or ethnicity (22).

Spanish women's football faces similar challenges to those in other European countries. A recent report by the Spanish Government's Ministry of Equality has shown that women's football—like other women's sports in Spain—continues to face obstacles such as players' work-life balance, job insecurity, incomplete professionalisation (only achieved at the highest level), lack of sponsorship and sexist attitudes (23). The report highlights that there have also been clear advances in recent years, particularly a better environment for younger footballers compared to that found by previous generations and greater public visibility for women's football. Proof of the growth in support for this sport in Spain is that, between 2019 and 2022, attendance records for women's football matches were broken, with the world record set at 91,553 fans who attended the Women's Champions League semi-final between Barcelona and Wolfsburg at the Camp Nou (24). High television ratings (at least for the national team) (25) and growing media coverage (1) also point to increased interest^4^. All of this translates into greater economic benefits for women's teams (27), thereby increasing their financial capacity. Despite persistent obstacles, it can be said that Spanish women's football is at the *avant-garde* of women's sport worldwide.

Of course, part of this progress is due to the undeniable advances in gender equality that Spain has made over the last two decades, both at an institutional and socio-cultural level (see next section). However, the role of female footballers' activism in these advances cannot be overlooked (23). Here, we understand activism as direct action to bring about social or political change (28). A clear example of this activism and its impact are the economic and labour demands of Spanish professional female players (agreement on minimum wage, maternity leave, professionalisation, etc.). In 2018, negotiations began to secure a collective agreement, and the players opted for non-confrontational strategies (such as collective bargaining and statements in the media). However, their demands were ignored by the clubs and various football organisations until a strike by the players (a confrontational strategy) forced an agreement in early 2020 (3). In view of the many successes achieved by Spanish women's football (both at club and national team level) and the increase in its popularity and support, the players demanded a renegotiation of the collective agreement in 2023. In this case, they requested an increase in the minimum wage and the complete disappearance of part-time contracts (which meant *de facto* recognition of their status as professional athletes^5^). Given the precedents, the players once again opted for a confrontational strategy, declaring two days of strike action to coincide with the start of the national women's league in September 2023. Finally, the employers' association representing the clubs accepted the players' demands, raising the minimum wage to €23,500 over the next three years (7).

These demands were not only key to start reducing the precariousness and job insecurity of female players, but also increased the capacity of Spanish women's football to achieve international success (as happened, particularly after the first strike): advances in its professionalisation strengthen the domestic league, which we know increases the chances of sporting success for national teams (29–31). However, although the athletes' activism strategies may be beneficial both for sport (in particular) and for gender equality (in general), these demands may also encounter resistance from public opinion. In fact, sport has traditionally been seen as a “neutral” space in political and ideological terms (32), which makes it necessary to explore the level of support for these demands, as well as the existence of rejection to them and its determinants. However, we know very little about the attitudes and preferences of Spanish citizens regarding the labour demands of female footballers.

Examining the attitudes towards these demands would advance the sociological study of sport in Spain and offer insights from a comparative perspective. Although we have quite a few studies on social attitudes and preferences towards athlete activism in other countries, most of them focus on the United States (8). The few exceptions that have addressed this issue in the European context—focusing on German men's football—have observed higher levels of support for athlete activism and less aversion to progressive causes compared to the North American findings^6^ (33, 34). These studies have also highlighted the relevance of the ideological correspondence between the athlete's demand and the political beliefs of the interviewee [(33, 34); see also Mudrick et al. (70)].

By examining social attitudes towards the activism of female footballers, we can assess whether the findings regarding men's football in Europe also apply to women's football. For the Spanish case, our expectation is that support for the economic and labour demands of female footballers will be high, in line with the high level of support that Spaniards show for gender equality (37)^7^. However, we also expect ideological identification and political partisanship to be a relevant factor in understanding the different attitudes and preferences of citizens in this regard, as the public debate took place in a very specific context, as detailed in the following section.

### 1.2 Feminism, anti-feminism and politics: Spanish women's football team and the Rubiales case

From 2018 to date, feminism and gender equality have been prominent topics in Spanish public debate. On 8 May of that year, historic demonstrations took place in the country's major cities, and more than 5 million Spaniards participated in the first national feminist strike in history. More mass marches took place across the country throughout 2018, 2019 and 2020 (before the pandemic), mobilising thousands of people, mainly women (39). These events can be framed within the global feminist wave generated by the *#MeToo* movement, but they were also a social response to the series of cases of sexual violence that occurred that year in Spain and their poor judicial treatment (40, 41). After the pandemic, social interest remained high, although with less mobilisation in the streets (mainly concentrated on the multiple demonstrations held every 8 May throughout the country). All these years of prominence and social mobilisation around this issue (between 2018 and 2023) had a profound effect on Spanish public opinion (42). This can be clearly seen in the IPSOS report: the number of people stating that gender equality was part of their daily conversations with family and friends doubled during this period (from 18% to 35%), and there was also a notable increase in the number of people identifying as feminists (from 44% to 53%). According to this data, Spain ranked as the European country with the highest level of support for feminism (37).

However, parallel to this development, a (growing) social trend against feminism also began to emerge in 2023 (39). In 2018, the percentage of respondents who considered that “too much” was being done to achieve equal rights between men and women in Spain was between 2% and 8% (depending on the area they were asked about); in 2023, the percentage of respondents who agreed with the statement “the promotion of women's equality has gone so far that men are now being discriminated against” rose to 53% (37, 43). Study No. 3428 by the CIS (44) also detected a high degree of agreement with this latter statement (39%, although lower than in the IPSOS report). Despite the contradictory nature of these trends, some studies have shown that the profile of support/rejection of gender equality is strongly influenced by factors such as gender, age and ideology (39), pointing to a growing polarisation around this issue. It is worth noting that, in Spain, polarisation has not been limited to gender equality but is part of a broader context of growing ideological polarisation (45), which is creating deep social divisions (46). However, the polarisation surrounding feminism and gender equality does not seem to have emerged as an extension of the polarisation surrounding other political issues: some authors point out that the rise in anti-feminist positions was a backlash against the social advances brought about by this social movement (47–49).

Among the various spaces and actors involved in this countermovement, one stands out: VOX. In the period 2018–2023, this radical right-wing party made the rejection of feminism and gender equality one of its political flagships (50, 51). We have evidence of their categorical rejection of feminism and its demands in their parliamentary speeches (52) and in their social media posts (53, 54). Not surprisingly, analyses of the content of the manifestos of the various Spanish parties show that VOX stands out as the only party that defends regressive policies on women and gender equality issues (55, 56). This stance has also given them electoral returns. In fact, in the Andalusian regional elections of December 2018 (the year in which the strong feminist mobilisation described above began), this political party won electoral representation for the first time. The work of Ramis-Moyano, Pasadas-del-Amo and Font (57) shows that rejection of feminism (along with territorial positions, rejection of immigration and authoritarianism) was one of the main factors that drove 11% of voters to choose this party's ballot in those elections. Since then, VOX has obtained election results of around 10%–15% in several elections, and other studies have confirmed and delved deeper into the influence of sexism and anti-gender equality stances among its electorate (47, 58).

It is not surprising, then, that this party uses high-profile cases to position its anti-feminist discourse^8^. This is what happened with the “Rubiales case”: the non-consensual kiss given by the president of the RFEF (Luis Rubiales) to Jenni Hermoso (a player of the national team) during the awards ceremony after the 2023 Women's World Cup. This case of sexism triggered a strong social and political outcry (4, 6): the hashtag *#SeAcabó* (“It's over”) went viral on Twitter, showing social support for the player and demanding accountability for the incident. Social pressure and press coverage of this case in the national and international media ultimately led to the resignation of the RFEF president. However, before his resignation, both the federation and Luis Rubiales himself attempted to defuse the crisis: first by downplaying the seriousness of the situation, and then by portraying the aggressor as a victim of “extreme feminism”. This strategy was accompanied by a discourse rejecting feminism and demands for equality, denying sexual and gender-based violence, and stigmatising the players as “childish” or “unprofessional” (5). This discourse was welcomed by some social sectors (particularly men), with whom VOX clearly aligned itself. In fact, although all political parties reacted quickly to the event, VOX reacted publicly 10 days after the event to denounce “the political and media witch hunt to which Mr Rubiales is being personally subjected”^9^ and to accuse “false feminism” (60). Not surprisingly, a survey at the time indicated that VOX voters were (by far) the least likely to condemn Luis Rubiales' sexist behaviour (61).

In line with what was argued in the previous section, we expect that this context of (growing) polarisation around gender equality in Spain will reinforce ideological identification as a relevant factor in understanding citizens' attitudes and preferences towards female footballers' labour demands. Given VOX's role in defending anti-feminist positions, we also expect partisanship to play a relevant role. Furthermore, we also expect gender to influence reactions to these demands, as we know that this is a relevant factor in support/rejection for feminism and gender equality (39); sexist and anti-feminist discourse also resonate much more strongly with right-wing men (47, 58). Finally, it should be noted that VOX's narrative of stigmatisation of female footballers was built around the “Rubiales case” and not around their economic demands, as the former was the most high-profile event. This meant that their narrative was closely linked to the success of the women's team in the international championship. Therefore, we also expect that the relevance of ideological identification, partisanship and gender will also be transferred to satisfaction with this sporting success, highlighting the negative predisposition towards this group of players beyond their achievements.

## 2 Methods

Our analysis draws on secondary data collected shortly after the Spanish Women's National Football Team won the FIFA World Cup in August 2023. This timing offers a unique opportunity to examine how gender and ideology shape public reactions to a landmark moment for women's sport in Spain. The survey provides nationally representative data that allow us to explore different dimensions of these reactions.

The following subsections describe the data source, the operationalisation of variables, and the analytical strategy. We first summarize the main characteristics of our data source and then detail the construction of dependent and independent variables and our analytical strategy.

### 2.1 Data

This study uses data from the October 2023 Barometer (Study No. 3423) of the Centro de Investigaciones Sociológicas (CIS) (9), a nationwide monthly survey designed to capture public opinion in Spain on political and social issues. The survey covers the Spanish population aged 18 and over and aims to represent the national socio-demographic structure by sex, age, region, and municipality size, using the Population and Housing Census of the National Statistics Institute (INE) as a reference.

The sample consists of 4,029 respondents drawn through a stratified random sampling design, combining landline (21.1%) and mobile (78.9%) telephone numbers. Within each stratum, quotas for sex and age were applied to ensure representativeness. Fieldwork was conducted between 2 and 6 October 2023 using computer-assisted telephone interviews (CATI).

The questionnaire included a thematic block on women's football (questions Q7–Q11), introduced shortly after the Spanish women's national team won the 2023 FIFA World Cup. These questions explore public attitudes towards the team's victory, the players' labour demands, and perceptions of gender differences in football.

### 2.2 Dependent variables

Our analyses rely on two dependent variables that capture distinct but complementary dimensions of public attitudes towards the Spanish Women's National Football Team and its mobilisation: support for the players' labour demands and satisfaction with Spain's World Cup win. The full wording of these items is presented in Supplementary Table 1 (Supplementary Materials).

The first dependent variable, support for the players' demands, measures respondents' agreement with the demands advanced by the footballers during their mobilisation in September 2023. Responses were recorded at a nominal level, with two categories: “yes” (support) and “no” (do not support). This binary operationalisation allows for a straightforward estimation of a logistic regression model, given that the outcome clearly distinguishes between respondents who endorse and those who reject the players’ demands.

**Figure 1:**
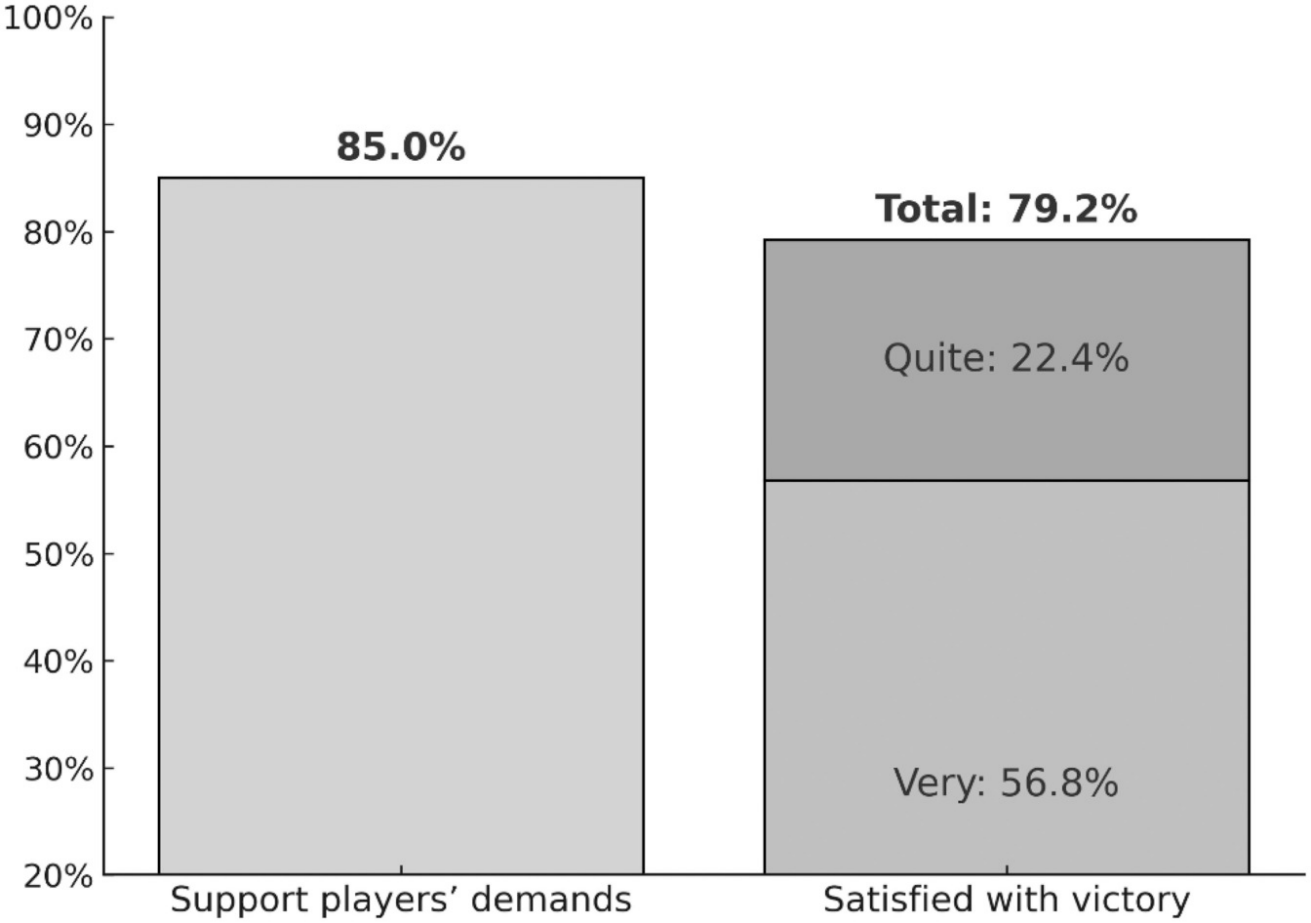
Proportion of respondents supporting the players’ demands and satisfied with the team’s victory.

The second dependent variable, satisfaction with the team's victory, captures respondents' emotional reactions to the sporting success of the Spanish women's national team. The survey question asks: “How satisfied are you with the Spanish national football team's World Cup victory?”. Responses were originally measured on a five-point Likert scale, ranging from “very satisfied” to “not at all satisfied” Figure 1.

Because most respondents reported being “very” or “quite satisfied,” the distribution of the satisfaction variable was highly skewed. To improve analytical clarity and model stability, we dichotomized the variable: respondents who declared themselves “very satisfied” were coded as 1, and all others as 0. This approach captures the strongest satisfaction with the team's success and reflects the main source of variability in the data.

### 2.3 Independent variables

To account for variation in both dependent variables, we included a common set of individual-level predictors that capture key demographic, socioeconomic, and political dimensions theoretically linked to attitudes towards the women's national football team and its mobilisation.

Specifically, the models include:

- Sex, coded as *1* *=* *female* and *0* *=* *male*.
- Age, grouped into ten-year intervals to account for non-linear life-cycle effects.
- Educational level, recoded into three categories: *primary or less*, *secondary*, and *higher education*.
- Ideological self-placement, measured on a *1–10 left–right scale*, treated as a continuous variable.
- Vote recall (July 2023), distinguishing between *Sumar, PSOE, PP, VOX, regionalist parties, other parties, non-voters,* and *no answer*.

These variables were selected on both theoretical and empirical grounds. From a theoretical perspective, demographic and political attributes such as gender, education, and ideology are consistently found to shape orientations towards gender equality, collective action, and national representation in sport. From an empirical standpoint, their inclusion follows the patterns observed in our descriptive analyses, where they exhibited the most substantive and statistically meaningful associations with the outcomes of interest.

Additional variables capturing contextual and status-related variation such as *municipality size*, *subjective social class identification*, and *occupation*, were also assessed in preliminary models. However, they did not reach statistical significance or alter the main effects and were therefore excluded from the final specifications for the sake of parsimony.

In the satisfaction model, we also included support for the players' demands as an independent variable. People who support the players are expected to feel more satisfied with the team's victory, since the achievement could be interpreted both as a sporting success and as a symbolic feminist victory (which, in turn, would confirm the moral and political stance of the interviewees). Empirically, preliminary analyses confirmed a strong and consistent association between support for the players and reported satisfaction, justifying its inclusion as a key predictor in the model.

### 2.4 Data analysis

Two binary logistic regression models were estimated to predict (1) *support for the players' demands* and (2) *satisfaction with the team's victory*. Each model includes socio-demographic and ideological predictors. To rule out potential multicollinearity, Variance Inflation Factors (VIF) were computed using an auxiliary OLS regression with the same set of predictors (68). VIF values ranged between 1.0 and 2.0 (tolerance between 0.49 and 0.97), indicating no significant collinearity among the independent variables.

For robustness, preliminary models including interaction terms between sex and other predictors were evaluated, but none reached statistical significance. The final specifications therefore report main effects only.

Marginal effects and predicted probabilities were computed to facilitate interpretation of significant predictors, allowing results to be expressed as changes in predicted probabilities rather than log-odds (69). This enhances the substantive interpretation of the findings and enables meaningful comparison across predictors measured on different scales. The formula and a detailed explanation of the model are presented in the Supplementary Material.

## 3 Results

This section presents the main findings from the inferential analyses. We first examine the determinants of *support for the players' demands*, followed by the factors associated with *satisfaction with the team's victory*.

Results are displayed through graphical representations of marginal effects and predicted probabilities derived from the logistic regression models. These figures show how sociodemographic and political variables influence each outcome, allowing for an intuitive interpretation of the direction and magnitude of effects. Each plot reports estimated marginal effects with 95% confidence intervals. For transparency and replication, the complete SPSS output tables—including coefficients, standard errors, and model fit statistics—are available in Supplementary Tables 4, 5 in the Supplementary Materials.

The analyses aim to determine whether attitudes towards the players' mobilisation and satisfaction with the team's success are primarily explained by sociodemographic characteristics (such as gender, age, and education) or by political and ideological alignments, reflecting broader patterns of value polarisation within Spanish society.

### 3.1 Support for the players’ demands

**Figure 2:**
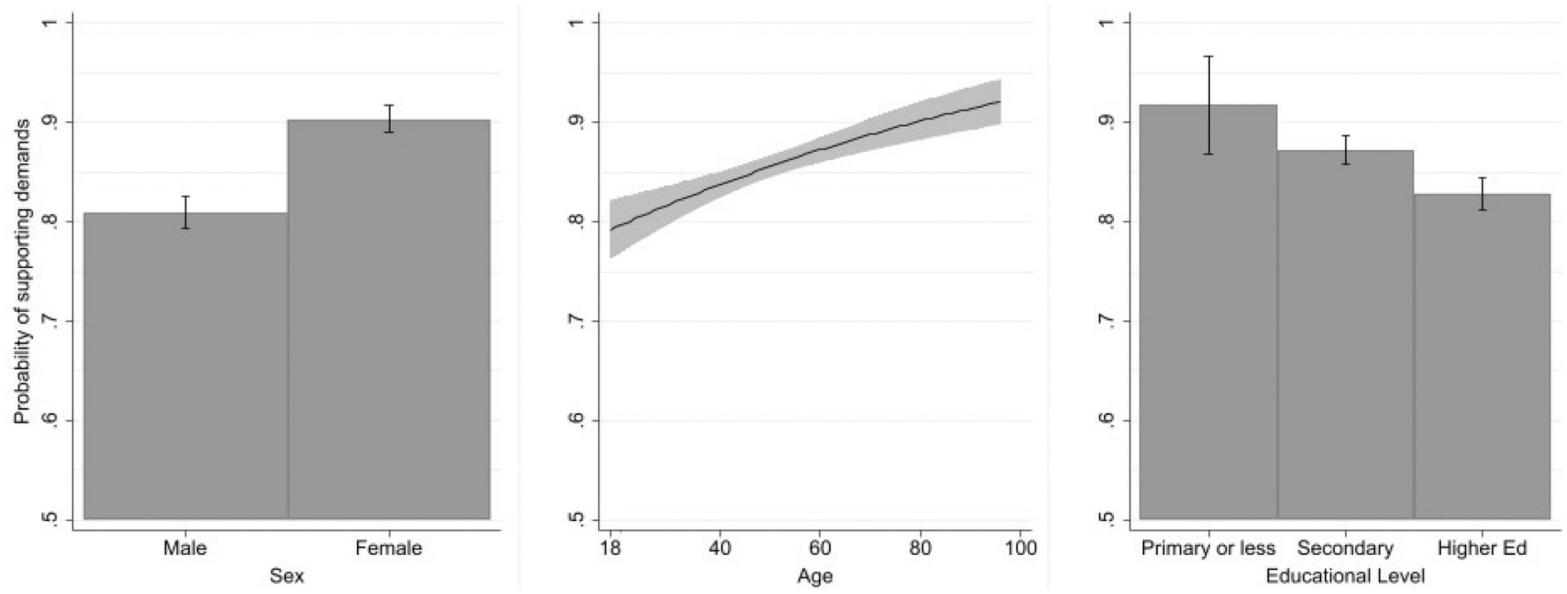
Predicted probabilities of supporting the players’ demands by sociodemographic variables. Bars and shaded areas represent 95% confidence intervals. Predicted values are based on binary logistic regression models including sex, age, and educational level, with all other variables held at their means.

Figure 2 displays the predicted probabilities of supporting the players' demands across sex, age, and educational level. Support is very high across all social groups, with estimated probabilities ranging from approximately 0.80 to 0.95. Nonetheless, the regression results reveal several statistically significant differences.

Sex is the most consistent predictor among the sociodemographic variables. The logistic model indicates that women are more than twice as likely as men to support the players' demands [B = 0.965, *p* < 0.001; Exp(B) = 2.63]. As shown in Figure 2 (left panel), the predicted probability of support among women exceeds 0.90, compared with roughly 0.80 for men. This robust gender gap aligns with expectations that women display stronger identification with demands framed around equality, recognition, and fairness in sport.

Age also shows a positive and significant association with support [B = 0.018, *p* < 0.001; Exp(B) = 1.018]. The predicted probability of endorsement rises gradually with age, from around 0.80 among the youngest respondents to nearly 0.90 among the oldest (Figure 2, middle panel). Although the magnitude of this increase is moderate, it suggests that older respondents may perceive the players' demands as more legitimate or consistent with broader fairness norms.

By contrast, educational attainment exhibits a statistically significant inverse relationship with support for the players' demands [*χ*² (2) = 19.60, *p* < 0.001]. As shown in Figure 2, the predicted probability of support declines gradually with education: from around 0.92 among respondents with primary education or less, to 0.88 among those with secondary education, and to approximately 0.83 among those with higher education. This result is somewhat counterintuitive, as higher education is typically associated with stronger egalitarian attitudes. The pattern, whereby lower educational levels express stronger support, likely reflects perceptions of fairness in the specific dispute rather than differences in abstract egalitarian values associated with education. A tentative explanation may lie in the particular context of professional football, where elite male players receive exceptionally high salaries—reversing the usual income hierarchy in which higher educational attainment corresponds to higher pay—although this hypothesis cannot be directly tested with the present data.

**Figure 3:**
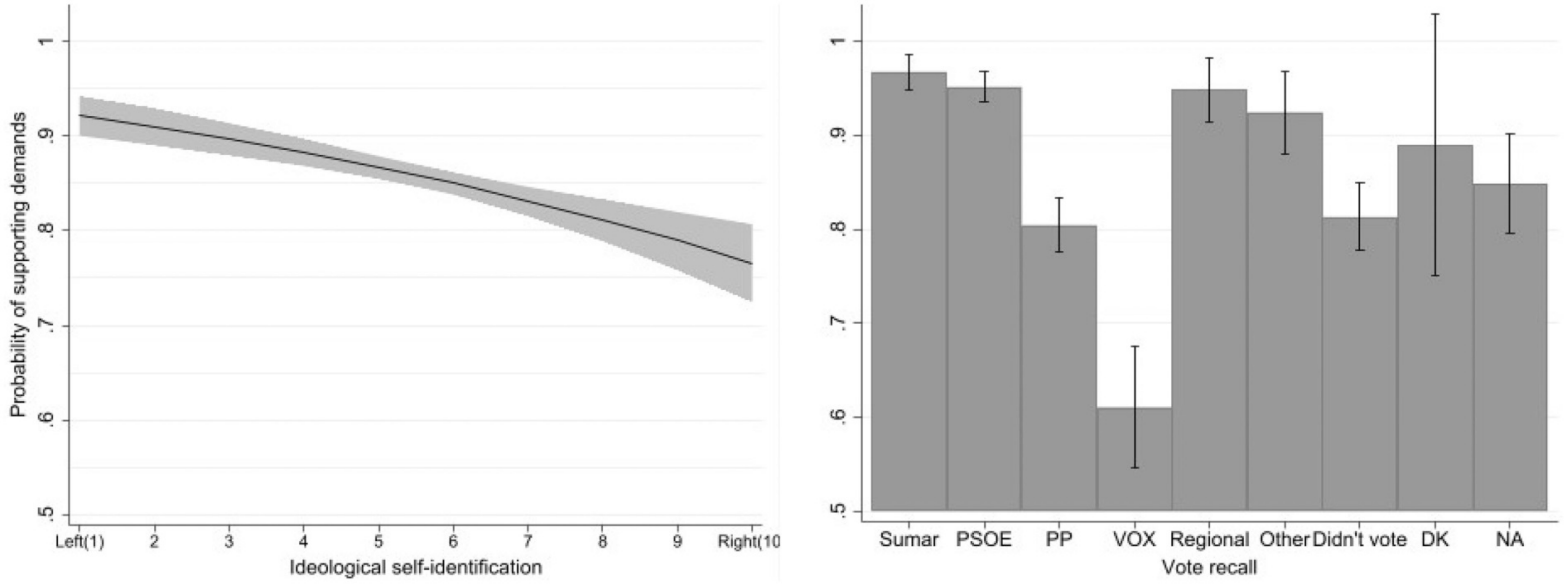
Predicted probabilities of supporting the players’ demands by political variables. Bars and shaded areas represent 95% confidence intervals. Predicted values are based on binary logistic regression models including ideological self-placement and vote recall (July 2023), with all other variables held at their means.

Figure 3 presents the marginal effects of political variables on support for the players' demands. Both ideological self-placement and vote recall are strong and statistically significant predictors.

Ideological orientation shows a clear and negative effect [*B* = –0.169, *p* < 0.001; Exp(B) = 0.845]. Support declines steadily along the left–right continuum: respondents identifying with the left exhibit near-unanimous support (predicted probabilities above 0.90), while those on the right fall closer to 0.70. This pattern underscores the ideological structuring of gender- and equality-related attitudes in Spain's political landscape, with right-leaning individuals expressing comparatively lower empathy towards the players' mobilisation.

Vote recall further reinforces this interpretation [*χ²* (8) = 163.47, *p* < 0.001]. Support is highest among voters of Sumar [Exp(B) = 7.47, *p* < 0.001], PSOE [Exp(B) = 4.87, *p* < 0.001], and regionalist parties [Exp(B) = 4.57, *p* < 0.001]. VOX supporters stand out as the only group significantly below the reference category [Exp(B) = 0.32, *p* < 0.001], confirming the ideological polarisation of responses. PP voters [Exp(B) = 0.94, n.s.] and non-voters [Exp(B) = 2.99, *p* = 0.002] occupy intermediate positions, while respondents who did not recall their vote do not differ significantly from the baseline.

These findings indicate that political ideology and partisan affiliation are the most powerful predictors of support for the players' demands, shaping attitudes far more sharply than sociodemographic characteristics. The results suggest that while support for the footballers was widespread, its intensity and meaning were clearly structured along political lines, reflecting the intersection of gender equality, identity, and ideology.

### 3.2 Satisfaction with the team's victory

**Figure 4:**
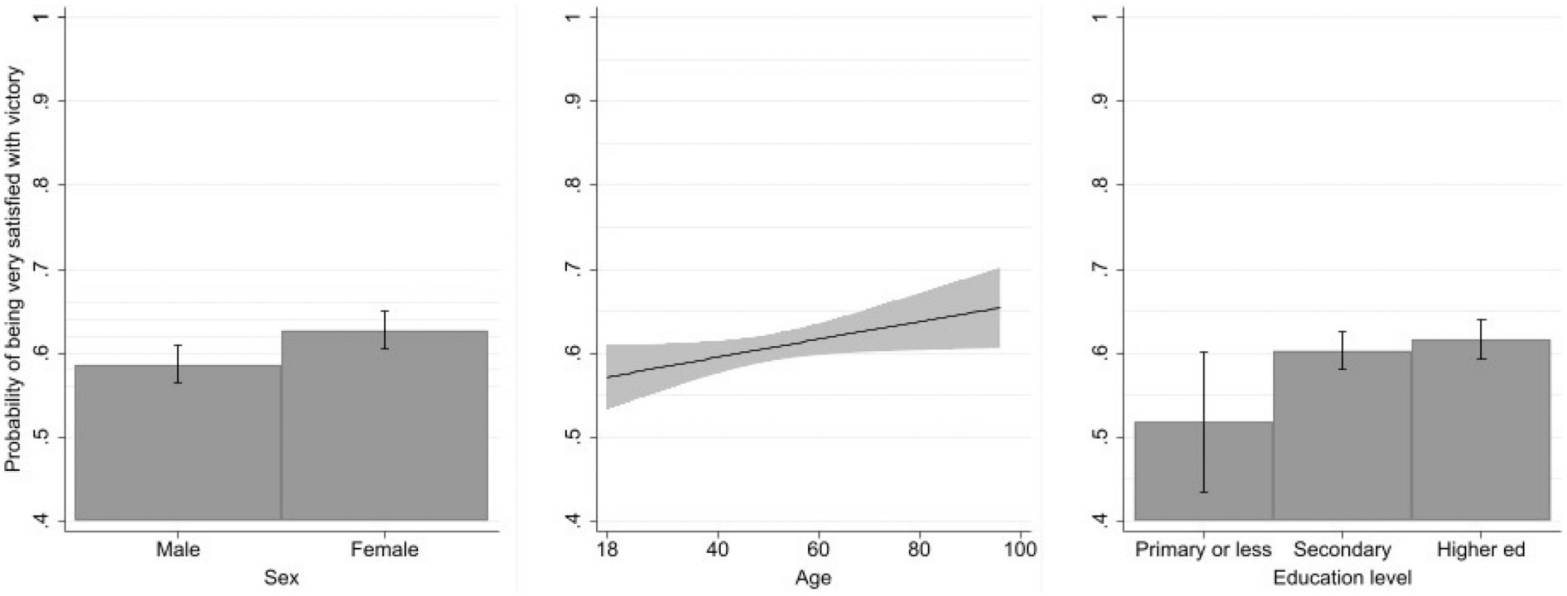
Predicted probabilities of being very satisfied with the team's victory by sociodemographic variables. Bars and shaded areas represent 95% confidence intervals. Predicted values are based on binary logistic regression models including sex, age, and educational level, with all other variables held at their means.

Figure 4 presents the marginal effects of the sociodemographic predictors included in the logistic regression model. The results show that gender, age, and education to a lesser extent, each contribute modestly but significantly to explaining satisfaction with the Spanish Women's National Football Team victory.

Women display a slightly higher predicted probability of being very satisfied than men, a difference that is statistically significant [Exp(B) = 1.209, *p* = 0.012], even after controlling for support for the players' demands. This finding, also evident in Figure 4 (left panel), indicates that female respondents were more likely to react very positively to the team's success.

A similar pattern is observed for age, which has a small but positive effect (*B* = 0.005, *p* = 0.044). The middle panel of Figure 4 shows a gradual increase in the probability of reporting high satisfaction among older respondents, possibly reflecting generational differences in the value attached to national sporting achievements.

The effect of education (right panel) is not statistically significant overall (*p* = 0.072), although respondents with primary education or less display significantly lower satisfaction than those with higher qualifications [Exp(B) = 0.681, *p* = 0.049] after controlling for support for the players' demands. Differences among the upper educational categories are minor, suggesting only a weak and inconsistent association between educational attainment and satisfaction with the team's success.

**Figure 5:**
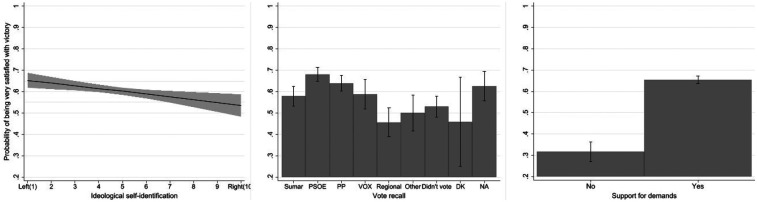
Predicted probabilities of being very satisfied with the team's victory by political variables. Bars and shaded areas represent 95% confidence intervals. Predicted values are based on binary logistic regression models including ideological self-placement, vote recall (July 2023), and support for the players’ demands, with all other variables held at their means.

Political orientation and support for the players' demands emerge as the strongest correlates of satisfaction, underscoring that reactions to the team's victory are structured by ideological and symbolic factors rather than by sporting considerations alone (Figure 5).

As with support for the players' demands, ideological self-placement shows a clear and statistically significant negative effect [*B* = –0.060, *p* = 0.004; Exp(B) = 0.942]. The predicted probabilities decline gradually from the left (≈0.65) to the right (≈0.50), indicating that individuals situated further to the right of the ideological spectrum are less likely to declare themselves very satisfied. This pattern suggests that even national sporting achievements are not entirely exempt from political meaning: left-leaning respondents may view the team's success as part of a broader narrative of women's empowerment, whereas right-leaning individuals may identify less strongly with that symbolism.

Vote recall reveals a more complex configuration [*χ*² (8) = 65.11, *p* < 0.001]. The regression coefficients show significantly higher odds of satisfaction among PSOE [Exp(B) = 2.01, *p* < 0.001] and PP voters [Exp(B) = 1.63, *p* < 0.001], both above the sample average. This suggests that the victory resonated across the electorate of Spain's two main national parties, with both gender-equality symbolism and national pride potentially contributing to their voters' satisfaction, albeit with different relative weights in each electorate. However, this interpretation remains tentative, as the available data do not allow us to directly assess the relative importance of these factors. In contrast, respondents who voted for regionalist parties [Exp(B) = 0.73, *p* = 0.086] display significantly lower levels of satisfaction, possibly reflecting a weaker affective connection to a Spanish national team victory among electorates with distinct territorial identities.

Finally, support for the players' demands stands out as the strongest and most significant predictor in the model [B = 1.451, *p* < 0.001; Exp(B) = 4.27]. Respondents who support the players have a predicted probability of being very satisfied of around 0.67, compared with only 0.30 among those who did not. This substantial difference confirms that reactions to the team's victory reflect not only a sense of sporting pride but also symbolic alignment with the players' cause and the broader issues it represented.

The logistic regression results show that satisfaction with the team's victory is partly shaped by support for the footballers' demands, which weakens the influence of traditional sociodemographic factors. Among Sumar and VOX voters, the expected differences in satisfaction largely disappear once support for the players' demands is considered, suggesting that their reactions are mediated by broader orientations towards feminism or anti-feminism. A similar, though smaller, pattern appears with education: respondents with lower educational attainment, who expressed the highest initial support for the players' demands, report comparatively lower satisfaction once that support is accounted for, whereas the negative association between higher education and support does not extend to satisfaction. Overall, these results indicate that identification with the players' cause is a key, though not exclusive, factor in shaping emotional responses to the team's success.

In summary, satisfaction with the women's team's victory is widely shared but clearly structured by political and attitudinal factors. Sociodemographic differences are modest, whereas ideology, partisanship, and especially support for the players' demands play a decisive role. These results suggest that while the event generated a broad sense of national pride, its emotional meaning varied across political constituencies, reflecting the intersection of sport, gender, and ideology in Spain.

## 4 Discussion and conclusions

In this article, we have addressed social attitudes and preferences towards women's football. Specifically, we have examined Spanish citizens' support for the players' labour and economic demands, and how this influenced attitudes towards the Spanish Women's National Football Team after its victory in the 2023 FIFA Women's World Cup. To do so, we have used data from Barometer No. 3423 of the Centro de Investigaciones Sociológicas (CIS) (9), the only data available on this subject, obtained less than two months after the national team's sporting success. This achievement provides a good context for study, as it not only received extensive media coverage (1), but was also immediately followed by two events that helped to place the players' working conditions at the centre of public debate (2). Firstly, the “Rubiales case” (4, 6) and, shortly afterwards, the strike by professional players to improve their working and economic conditions (7). This context of ongoing conflict between the players and the main Spanish football institutions turned the sporting victory into a symbol of the struggle for gender equality, but also into an object of cultural and political dispute.

Our work contributes directly to advancing the understanding of the cultural dynamics that shape attitudes towards women's sport and raises new questions for the sociology of sport. First, this is (to the best of our knowledge) the first study to address the level of support for activism among European female athletes, as well as its determinants. Previous studies have addressed this issue mainly in the North American context (8), and the few studies focused on the European context have addressed men's football (33, 34). In line with the findings of these latter studies, our results show that activism by female athletes in Europe is mostly well received, which differs from what has been observed in the North American context (32). Regression models also show, in line with these previous studies, the influence of ideological correspondence between the athlete's demand and the political beliefs of the interviewee on support for these demands (as discussed below). However, we highlight here the finding that points to gender as a key variable: women support the demands of female footballers much more than men, regardless of their ideology and voting preferences. This may have to do with the close relationship between gender equality and the case of activism analysed [see (62)], but the work of Müller et al. (34) has already shown that men are less supportive of progressive causes (regardless of their connection to gender equality).

Furthermore, our results show a positive relationship between age and support for the demands, unlike the findings of European studies on men's football [(34); but see (62)]. This result is counterintuitive, because one would expect younger cohorts (particularly women) to be more supportive of these demands (39). However, we know that the youngest cohorts also show the highest levels of rejection of gender equality (particularly among men) [(37); see also (63)]. This could be causing these cohorts to have less support for the demands than older cohorts on average. Future work on female athlete activism will need to corroborate whether our results are exceptional or whether, on the contrary, they can be extended to other European countries or other women's sports.

Secondly, our work also contributes to the study of women's sport in Spain, as the degree of support for the Spanish Women's National Football Team and, more generally, for women's football in Spain had not yet been explored. Our results point, in general, to a high level of support for women's football (both for its demands and for its sporting successes). We find this result in spite of the context analysed: in 2023, there was a noticeable increase in attitudes rejecting feminism for having gone “too far” (37, 39, 44), and the victory in the Women's World Cup that year was overshadowed by the “Rubiales case” and the anti-feminist discourse that emerged to defend the former president of the RFEF (5). One potential explanation for this striking result may be that, in response to the salience of a gender equality advocacy event, public opinion shifts (even if only temporarily) towards more progressive positions, as shown in the experiment by Alexander et al. (64). In this way, the prominence of the conflict between the players and the major football institutions may have increased social support for the players. This is only one interpretation, but to better understand this result future research should examine, for example, the extent to which the Spanish Women's National Football Team is associated with the defence of feminist and gender equality causes, or the extent to which their claims are perceived as fair or necessary.

Thirdly, our work also contributes to the study of the intersection between the sociology of sport and political sociology. In fact, both ideological orientation and partisan affiliation are the most relevant variables for understanding attitudes towards women's football. Although levels of rejection of the demands and sporting success of Spanish players are low, upon examination we observe that, in both cases, rejection is significantly higher among right-wing individuals. Being a VOX voter also significantly increases rejection of the players' labour demands (although this is not significant in explaining the reduction in satisfaction with victory). These results mirror the pattern identified by Müller et al. (34) regarding the lower support among right-wing men for sports activism promoting progressive causes, as is also the case here. What is striking is that this profile is also reflected in lower levels of satisfaction with the sporting success of the women's team, even when support for the players' labour demands is controlled for in the regression model. This suggests that, beyond the demands themselves, this profile is highly predisposed to view women's football negatively. This is in line with the sociodemographic profile that usually takes a negative stance on feminism and gender equality in Spain (39) and which has found political representation in VOX, a radical right-wing party that makes anti-feminism one of its main flags (47, 57, 58).

As we have indicated, this party stood out during the “Rubiales case” by supporting the aggressor and reinforcing the stigmatisation of the players as “childish” or “unprofessional” (5, 60). It is therefore not surprising that, among this party's voters, we find significantly less support for the players' demands. Although VOX voters' satisfaction with the sporting success is also lower than that of voters from other parties, this result is not statistically significant because it is largely captured by the inclusion in the regression model of the variable on rejection of the players' demands. With our analytical strategy, we cannot attribute causality to VOX's anti-feminist discourse on the rejection of women's football and its demands, but our results do highlight the fact that attitudes towards women's sport are strongly influenced by political debates. This was already pointed out by Pfister (11), who noted the potential backlash against women's sport due to the fear generated by the perception that it challenges established hierarchies and patriarchal structures [on this, see (65)]; therefore, anti-feminist discourse that allows these fears to be channelled (as well as the actors who reproduce them) poses an added challenge for women's sport.

Our study is not without limitations. The survey data offer a static snapshot and do not capture how public attitudes may have evolved over time—for instance, before and after the team's sporting success, or in the period since. In addition, because data were collected shortly after Spain's World Cup victory, when media coverage and emotional responses were at their peak, levels of support and satisfaction may have been temporarily amplified by the exceptional visibility of the event. Moreover, this article focuses on public attitudes regarding women's football in Spain, a context that is distinctive for its high visibility of women's sport and broad public support for gender equality (37). Future research should extend this line of enquiry both temporally, to assess changes in public attitudes over time, and geographically, to compare perceptions of women's football across different national contexts.

The main limitation of the study, however, lies in the unavailability of some potentially relevant variables for inclusion in the regression models, as they were not collected in the original survey. This is particularly pertinent to the model explaining satisfaction with the team's victory, whose explanatory power is modest (*R*² = 0.13). Variables such as national sentiment—known to shape reactions to the national team (66)—, interest in football, or explicit support for gender equality could have enhanced the explanatory capacity of the models [see (62)]. To partially address these gaps, we employed several indirect strategies. For example, the vote recall variable was disaggregated to include “regionalist parties”, which we used as an indirect indicator of weaker Spanish national sentiment, consistent with research showing that these parties attract voters more attached to regional identities (67). Similarly, in the satisfaction model, we used support for the players’ labour demands as an indirect indicator of gender equality attitudes. Recognising these limitations, future research should incorporate more direct measures of these constructs to deepen understanding of public attitudes and preferences towards women's football and the broader struggle for gender equality in sport.

Despite these limitations, this study provides novel empirical evidence on how social and political factors shape public attitudes towards women's football in Spain. Our findings show that both support for the players' demands and satisfaction with the team's success are structured by gender, ideology, and alignment with the players' cause, rather than by sociodemographic factors alone. These findings highlight the value of examining sport as both a cultural and political arena through which wider societal debates about equality, identity, and representation are negotiated.

## Data Availability

The original contributions presented in the study are included in the article/Supplementary Material, further inquiries can be directed to the corresponding author.

## Footnotes

1 Real Federación Española de Fútbol (RFEF), the highest governing body of football in Spain.

2 However, as Pfister (11) points out, there have also been some (minority) sports with a greater female presence, distinguishing those sports that are predominantly feminine from those that are predominantly masculine.

3 In fact, in some European countries, women's football was banned in the two decades following the Second World War (17).

4 Despite unequal treatment compared to the men's national team (26).

5 The status of “professional sport” was formally recognised by the Comisión Directiva of the Consejo Superior de Deportes (CSD) very recently, on 15 June 2021. This came after the approval of the collective agreement that was in force until that time (in 2020).

6 Probably because the average US fan (on whom these studies are based) is very conservative, displaying strong nationalism, as well as high support for the US military and individualistic ideas of economic success (35, 36).

7 This expectation is also in line with the results shown by Wicker and Cunningham (38) in their analysis of Europeans' attitudes towards gender equality in sport: a better gender climate at national level predicts a better attitude towards female sporting role models.

8 In line with what some studies have pointed out about radical right-wing parties adapting to the social context and seeking out ‘niches’ in which their ideological discourse can stand out better (59).

9 The English translation is our own.
