# Supplementary material for: Pride or backlash? Public attitudes towards the Spanish women’s national football team
Source: Front Sports Act Living. 2025 Dec 2;7:1727889. doi: 10.3389/fspor.2025.1727889 (PMC12705638; doi:10.3389/fspor.2025.1727889)
Supplement: Supplementary file 1 [file Datasheet1.docx]

Supplementary Material

# Table S1. Questions analyzed in the study, included in the CIS Barometer of October 2023 (Study No. 3423)

# The items are part of a broader module on attitudes toward women’s football and women’s sport.

| Question | Answer options |
| --- | --- |
| Q7. Now I am going to ask you a few questions about women's football. How satisfied are you with the Spanish national football team's World Cup victory? Are you very satisfied, quite satisfied, not very satisfied, or not at all satisfied with the victory? | Very satisfied Quite satisfied Neither satisfied nor dissatisfied Not very satisfied Not at all satisfied Don’t know No answer |
| Q10. As far as you know, do you think that the demands being made by women footballers should be supported? | Yes No Don’t know No answer |

# Source: Authors’ elaboration based on data from Centro de Investigaciones Sociológicas (CIS, 2023).

# Table S2. Technical details of the CIS Barometer of October 2023 (Study No. 3423)

| Item | Description |
| --- | --- |
| Scope | National (Spain) |
| Population | Residents of Spain aged 18 years and older (both sexes) |
| Sample size | Designed: 4,000 interviews; Completed: 4,029 interviews |
| Sampling method | Stratified random sampling of fixed and mobile telephone numbers (21.1% landlines, 78.9% mobiles), with quotas by sex and age |
| Geographical stratification | 17 autonomous communities and 2 autonomous cities, crossed by 7 habitat size categories |
| Number of sampling points | 1,220 municipalities and 50 provinces |
| Weighting | Weighted to correct for unequal sampling rates across regions (variables PESO and PESOCCAA) |
| Margin of error | ±1.6% for a confidence level of 95.5% (two sigmas, P = Q = 0.5) |
| Mode of data collection | Computer-assisted telephone interviewing (CATI) |
| Fieldwork dates | 2–6 October 2023 |
| Source | Centro de Investigaciones Sociológicas (CIS), Barómetro de Octubre 2023 (Estudio nº 3423). Available at: https://www.cis.es/es/detalle-ficha-estudio?codEstudio=3423 |

# Source: Authors’ elaboration based on CIS (2023).

# Table S3. Descriptive statistics of the main variables used in the analysis

| **Variable** | **Categories / Scale** | **Valid N** | **% (Valid)** |
| --- | --- | --- | --- |
| Satisfaction with the women’s national team victory (Q7) | Very satisfied Fairly satisfied Neither satisfied nor dissatisfied Not very satisfied Not at all satisfied  DK/NA | 4029 | 56.8 22.4 7.0 5.5 4.5  3.8 |
| Dichotomized satisfaction (dependent) | 1 = Very satisfied 0 = All others | 3875 | 59.0 (Yes) 41.0 (No) |
| Support for players’ demands (Q10) | Yes No  DK/NA | 4029 | 76.4 12.6  11.0% |
| Sex | Male Female | 4029 | 51.5 48.5 |
| Age | Continuous (18–96 years) | 4029 | Mean ≈ 49 years |
| Education level | Primary or less Secondary Higher education | 3983 | 5.1 46.7 48.2 |
| Ideological self-placement (1–10) | 1 = Left … 10 = Right  DK/NA | 4029 | Mean ≈ 4.8  4.7% |
| Vote recall (2023 General Election) | Sumar PSOE PP VOX Regionalist parties Other parties Did not vote No response No answer | 4029 | 13.3 25.0 23.9 7.7 5.3 3.7 13.7 0.9 6.5 |

# Regression models specification

# Binary logistic regression models were estimated to predict (1) *support for the players’ demands* and (2) *satisfaction with the team’s victory*. The general form of the model is:

#

$$\ln\left( \frac{\boldsymbol{p}_{\boldsymbol{i}}}{\mathbf{1-}\boldsymbol{p}_{\boldsymbol{i}}} \right)\mathbf{=}\boldsymbol{\beta}_{\mathbf{0}}\mathbf{+}\sum_{\boldsymbol{j}\mathbf{=1}}^{\boldsymbol{k}} \boldsymbol{\beta}_{\boldsymbol{j}}\boldsymbol{X}_{\boldsymbol{j}\mathbf{,}\boldsymbol{i}}\mathbf{,}$$

# where $\boldsymbol{p}_{\boldsymbol{i}}$denotes the probability of the event of interest (1 = support for the players’ demands; 1 = very satisfied with the victory), and $\boldsymbol{X}_{\boldsymbol{j}\boldsymbol{,}\boldsymbol{i}}$are the explanatory variables. The exponential of each coefficient, Exp(B), represents the odds ratio associated with a one-unit increase in the predictor (or with each category, relative to the reference, for categorical variables).

# All models were estimated in IBM SPSS Statistics (version 29), and marginal effects and predicted probabilities were computed in Stata (version 17) for interpretation and visualization.

# Table S4. Binary logistic regression predicting support for the players’ demands

# (Dependent variable: Support for players’ demands = 1)

| **Predictor** | **B** | **SE** | **Wald** | **p** | **Exp(B)** |
| --- | --- | --- | --- | --- | --- |
| Sex (1 = female) | 0.965 | 0.116 | 69.16 | <.001 | 2.63 |
| Age | 0.018 | 0.004 | 23.93 | <.001 | 1.02 |
| Education (ref. = Secondary) |  |  | 19.60 | <.001 |  |
| Primary or less | 1.040 | 0.404 | 6.63 | .010 | 2.83 |
| Higher education | 0.444 | 0.113 | 15.46 | <.001 | 1.56 |
| Vote recall (ref. = Non-voters) |  |  | 163.47 | <.001 |  |
| Sumar | 2.011 | 0.328 | 37.56 | <.001 | 7.47 |
| PSOE | 1.584 | 0.225 | 49.62 | <.001 | 4.87 |
| PP | –0.061 | 0.166 | 0.14 | .714 | 0.94 |
| Vox | –1.128 | 0.198 | 32.60 | <.001 | 0.32 |
| Regionalist parties | 1.519 | 0.389 | 15.20 | <.001 | 4.57 |
| Other parties | 1.097 | 0.358 | 9.38 | .002 | 2.99 |
| No recall | 0.654 | 0.771 | 0.72 | .396 | 1.92 |
| No answer | 0.273 | 0.263 | 1.08 | .298 | 1.32 |
| Ideological self-placement (1–10) | –0.169 | 0.031 | 29.94 | <.001 | 0.85 |
| Constant | 0.808 | 0.250 | 10.42 | .001 | 2.24 |

Model fit: χ²(13) = 672.15, p < .001; Nagelkerke R² = .32; % correctly classified = 86.6%.

**Table S5. Binary logistic regression predicting high satisfaction with the team’s victory**

(Dependent variable: Very satisfied = 1)

| **Predictor** | **B** | **SE** | **Wald** | **p** | **Exp(B)** |
| --- | --- | --- | --- | --- | --- |
| Sex (1 = female) | 0.190 | 0.076 | 6.25 | .012 | 1.21 |
| Age | 0.005 | 0.002 | 4.07 | .044 | 1.01 |
| Education (ref. = Secondary) |  |  | 5.26 | .072 |  |
| Primary or less | –0.385 | 0.195 | 3.88 | .049 | 0.68 |
| Higher education | 0.064 | 0.077 | 0.68 | .409 | 1.07 |
| Vote recall (ref. = Non-voters) |  |  | 65.11 | <.001 |  |
| Sumar | 0.218 | 0.149 | 2.13 | .144 | 1.24 |
| PSOE | 0.696 | 0.134 | 27.09 | <.001 | 2.01 |
| PP | 0.488 | 0.138 | 12.47 | <.001 | 1.63 |
| Vox | 0.258 | 0.186 | 1.91 | .167 | 1.29 |
| Regionalist parties | –0.317 | 0.184 | 2.94 | .086 | 0.73 |
| Other parties | –0.125 | 0.211 | 0.36 | .551 | 0.88 |
| No recall | –0.304 | 0.471 | 0.42 | .518 | 0.74 |
| No answer | 0.426 | 0.194 | 4.85 | .028 | 1.53 |
| Ideological self-placement (1–10) | –0.060 | 0.021 | 8.43 | .004 | 0.94 |
| Support for players’ demands (1 = yes) | 1.451 | 0.122 | 141.63 | <.001 | 4.27 |
| Constant | –1.224 | 0.211 | 33.73 | <.001 | 0.29 |

Model fit: χ²(14) = 340.33, p < .001; Nagelkerke R² = .13; % correctly classified = 67.1%.
